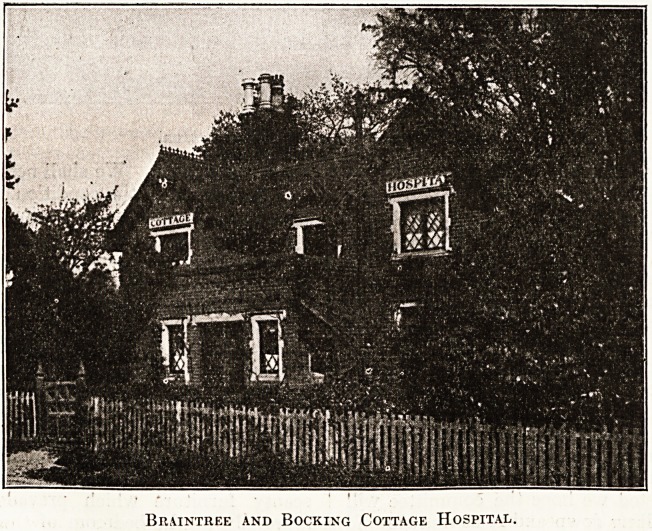# Reports on Hospitals of the United Kingdom

**Published:** 1913-11-22

**Authors:** Henry Burdett


### REPORTS ON

### Hospitals of the United Kingdom.

By SIR HENRY BURDETT, K.C.B., K.C.V.O.

SERIES III.

### ESSEX COUNTY HOSPITAL, COLCHESTER.

This hospital was established in 1820. We inspected it in 1896 and again in 1910, and on the latter occasion we made in our report certain suggestions, which included the provision of a new and up-to-date mortuary, of increased and better accommodation for the sisters and nursing staff, and of adequate larder accommodation for the food. Some of these suggestions have been carried out, but there is still an urgent need for the immediate provision of a well-ventilated, hygienic, and ample larder. It is not necessary for us to go at present into the condition of the hospital as we found it on the day of our third inspection, October 20, 1913.

A Nurses' Home was added in 1898, and a new children's ward was built and opened in 1908. Further additions, made in 1912, include a new isolation block, and a new pathological and mortuary section. The hospital contains 100 beds, of which eighty-three, on an average, are occupied. The new pathological and mortuary block is a welcome addition, the former section being one more proof of the vigour and energy with which the higher branches of medical work are being pursued, as they should be pursued, in a hospital of this importance and size. The new isolation block is, too, most welcome, and now that it has been completed we hope the committee will lose no time in pulling down and removing the old isolation block, thus opening out the hospital buildings, and at the same time make material improvements by re-arranging the grounds around the hospital.

Lady Pearson is to be congratulated and thanked for the children's ward, which, at the present time, is one of the most attractive and, from the curative point of view, one of the most fruitful and valuable departments of hospital work. We were very glad to notice the promising additions that were being made in the accommodation for the treatment of patients in the open air, a most important feature of the hospital, and one which we hope will be developed to the utmost possible extent. As to the wards, we found that the staff, who were mainly responsible for the efficiency and condition of the children's ward, and for the other ground-floor ward, were not available from illness or from some other cause. We shall not, therefore, express any opinion as to the condition in which we found them or make any suggestions, as we had an opportunity of going into the matter with the matron, who kindly accompanied us through the hospital. The upper-floor wards and all their offices were in excellent order throughout, and we congratulate the sisters and all who have contributed to this efficiency upon the smart and well-ordered appearance which they presented, in marked contrast to the state of affairs that at one time prevailed in this hospital.

Essex County Hospital, Colchester.

Whoever looks after the furnishing and equipment of the matron's and nurses' accommodation might usefully direct their special attention to the scanty furniture which prevails in places, and especially in the bedroom of the matron. Speaking generally we should say that this hospital was, as a whole, never in a better position than it is to-day, and that it gives a maximum of useful service and aid to the people of all classes who are dependent upon it in time of illness. The out-patient department of a hospital of this character is one which we expect speedily to disappear in favour of a casualty and consultation branch, but it may be well to mention that there appear to be no regular and enforced hours for the attendance of the medical staff, and that, as a consequence, patients are kept waiting—a thing which ought not to happen in a well-organised hospital of this type.

WHAT THIS HOSPITAL NEEDS.

For some years past it has been urged and demonstrated to repletion that the costly character of the modern work done, its importance to the community and the altered conditions under which an up-to-date hospital does its work at the present time, all tend to increase the cost of working and maintenance. This hospital has need of an increased income of some £2,000 a year. As we pointed out in 1910, it has now organisations representing the working classes, the women and teachers of Colchester, and other bodies. Having regard to the small sum raised in annual subscriptions, and to the general condition of the revenue account, we are of opinion that the time has come when energetic action should be taken to enlist as chairman some gentleman of position and influence, who would devote sufficient time and whole-hearted service to the direction of this hospital's affairs, and to drive home its claims and needs to the community, so that the present shortness of funds may speedily disappear. In this connection it was remarked to us in the course of our visit that Colchester is a "still-born place." May not the non-progressive character of the revenue and contributions have some other and deeper cause? It would be well for those responsible for the administration of this hospital to hold a careful inquiry, and to make up their minds whether or not they can reorganise the means which they at present employ for the raising of funds and for the general superintendence of affairs. They should then be in a position to come to some decision as to whether the course followed at other county hospitals might not be usefully adopted at Colchester?

We find that where a trained, experienced, and energetic administrator has been appointed as superintendent and secretary to a hospital of this character, at an adequate rate of remuneration, that modern methods have been introduced with encouraging results so far as an adequate increase in the funds, a sounder administration of the hospital in every department, and a more widespread popularity are concerned.

### THE BRAINTREE AND BOCKING COTTAGE HOSPITAL.

THIS charming little hospital was established in 1871, twelve years from the date of the opening of the first cottage hospital at Cranleigh. We visited it on October 18, 1913, and were charmed with it, from the circumstance that here the original ideas of the late Mr. Albert Napper, the founder of these institutions, have been faithfully carried out and his plans continued for upwards of forty years. It has a roomy porch or ante-chamber, which is tastefully fitted up, and over which we shall hope to see before long an open-air balcony provided for the outdoor treatment of suitable cases, which would give this attractive example of an original type a flavour of up-to-dateness and at the same time add to its practical usefulness. There is no pretension whatever about this institution or its system of management. Everything is of the simplest, and yet the buildings contain most things that are calculated to promote the welfare, comfort, and speedy recovery of the patients. There is a tiny operation theatre with somewhat primitive fittings, where some surgeons of eminence have operated and where good surgical work has been and no doubt will yet be done. The wards are simple, the beds and equipment are good, though we should like to see a hair mattress substituted for the one flock bed they contain. There is a bathroom of the smallest proportions, where a hot bath can be obtained under original conditions, which must interest the visitor in proportion to his knowledge of hospital work, whilst the other sections are adequately provided for in the most simple and practical manner.

BRAINTREE AND BOCKING COTTAGE HOSPITAL.

Miss Torn, a Black John nurse, has been in charge for some years, and seems to be just the right woman in the right place. She is evidently resourceful, intensely interested, thoroughly well trained, and is a most efficient matron. It was a pleasure to meet her, to be taken round by her, and to witness the interest and enthusiasm she has in everything connected with this exceedingly attractive building of a somewhat ancient type. We hope the present buildings may be permanently preserved, as has been done at Cranleigh, should the time ever arrive for the erection of a larger and more modern cottage hospital in the village. Meanwhile it is an interesting and creditable fact that good management has resulted in savings which represent an investment fund of nearly £1,500.

It is a pleasure to note, too, that the honorary secretaries who look after this little hospital (Miss Courtauld and Mr. W. J. Courtauld) are members of a family who have done so much during the last fifty years for the people of Halstead and the neighbouring villages. When we sum up all the splendid gifts and the resulting comfort with the opportunity for improved health and enjoyment which their provision has brought to the people of Halstead especially, it fills us with regret that the whole atmosphere and attitude of the residents in this place should not have changed for one which would better exhibit the intelligence and a much higher standard of individual personal responsibility on the part of Halstead people than they seem capable of under existing conditions. But the inhabitants of Bocking more often show much greater intelligence, and have also a higher sense apparently of personal responsibility and public duty. So Bocking has often left Halstead behind, as is fair and right.

**Halstead and Cromer Cottage Hospitals will be reported on next week.**

## Figures and Tables

**Figure f1:**
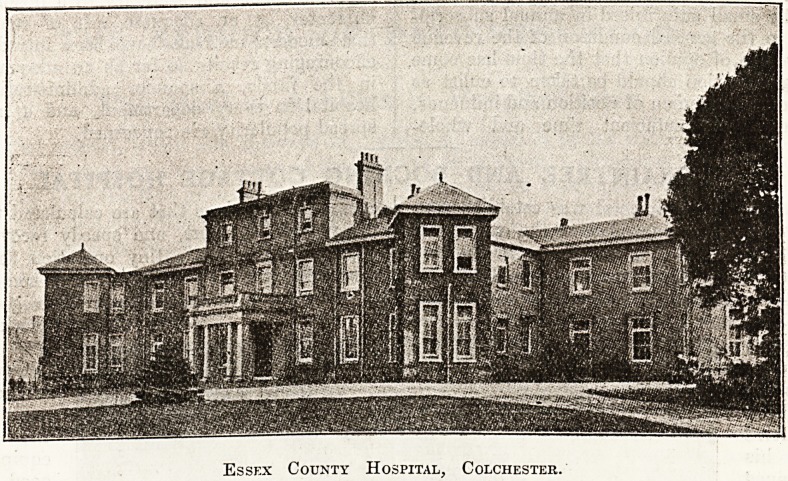


**Figure f2:**